# A Longitudinal Study of Physical Function Factors Related to Lower Limb Circumduction During Gait in Acute Stroke Patients with Hemiparesis

**DOI:** 10.3390/s25237309

**Published:** 2025-12-01

**Authors:** Ryosuke Shibuya, Yusuke Sekiguchi, Keita Honda, Midori Miyagi, Dai Owaki, Mitsuhiro Hayashibe, Satoru Ebihara

**Affiliations:** 1 Department of Rehabilitation Medicine, Tohoku University Graduate School of Medicine, 1-1 Seiryo-machi, Aoba-ku, Sendai 980-8574, Japan; keita.honda.d2@tohoku.ac.jp (K.H.); midori.miyagi.b5@tohoku.ac.jp (M.M.); satoru.ebihara.c4@tohoku.ac.jp (S.E.); 2 Department of Rehabilitation, National Hospital Organization Sendai Medical Center, Miyagino 2-11-12, Miyagino-ku, Sendai 983-8520, Japan; 3 Research Management Center, Organization for Research Promotion, Tohoku University, 2-1-1 Katahira, Aoba-ku, Sendai 980-8577, Japan; yusuke.sekiguchi.b2@tohoku.ac.jp; 4 Graduate School of Dentistry, Tohoku University, 4-1 Seiryo-machi, Aoba-ku, Sendai 980-8575, Japan; 5 Department of Rehabilitation, Kumamoto Health Science University, 325 Izumi-machi, Kita-ku, Kumamoto 861-5533, Japan; 6 Neuro-Robotics Laboratory, Department of Robotics, Graduate School of Engineering, Tohoku University, Sendai 980-8579, Japan; owaki@tohoku.ac.jp (D.O.); hayashibe@tohoku.ac.jp (M.H.)

**Keywords:** circumduction, acute stroke, abnormal gait pattern, longitudinal change

## Abstract

**Highlights:**

**What are the main findings?**

- Paretic femoral abduction and pelvic hike angles significantly decreased over time in acute stroke patients.
- Reduced femoral abduction correlated with paretic ankle motor recovery, while reduced pelvic hike correlated with paretic knee motor recovery.

**What are the implications of the main finding?**

- Femoral abduction and pelvic hike are distinct adaptive strategies, not a single “circumduction” pattern as often assumed.
- Compensatory movements in acute stroke should be re-evaluated as adaptive phenomena during recovery, not merely as abnormal movements.

**Abstract:**

Circumduction gait in stroke patients, a compensatory movement involving pelvic hike and femoral abduction, increases energy cost. However, longitudinal studies on its mechanism during the acute phase are lacking. This study longitudinally investigated changes in the paretic femoral abduction angle during gait in acute stroke patients and identified related factors. Twenty-two stroke patients were assessed twice: at gait initiation and 10–14 days later. Gait kinematics during a 3 m walk were measured using a depth sensor, and physical functions (SIAS) were evaluated. Changes were analyzed using paired *t*-tests and correlation analyses. Spatiotemporal parameters improved significantly. Kinematically, paretic femoral abduction (*p* = 0.049) and paretic pelvic hike (*p* = 0.025) significantly decreased, while maximum paretic knee flexion during swing (*p* = 0.026) increased. The decrease in femoral abduction correlated positively with the decrease in pelvic hike (r = 0.55) and negatively with the improvement in paretic ankle motor function (SIAS) (ρ = −0.49). The decrease in pelvic hike correlated negatively with the improvement in paretic knee motor function (SIAS) (ρ = −0.43). These results suggest that in acute stroke patients, the recovery of paretic ankle and knee motor functions leads to a reduction in compensatory femoral abduction and pelvic hike, respectively. This study provides insights for re-evaluating compensatory movements as an adaptive phenomenon during recovery, not merely as abnormal movements.

## 1. Introduction

Stroke-related hemiparesis impairs gait through deficits in lower-limb sensation and motor control, reducing daily activity and health-related quality of life (QOL) [1,2,3,4,5]. Elevated metabolic cost is a key contributor, and so-called circumduction has been implicated as an inefficient yet common compensation [4,5,6,7]. Rather than a single pattern, circumduction is typically expressed through two surrogate kinematic behaviors during paretic swing: thigh (femoral segment) abduction and pelvic hike [8]. In this study, we define thigh abduction angle as the angle between the global vertical and the thigh segment, and pelvic hike angle as the angle between the horizontal and the line connecting both anterior superior iliac spines; positive values indicate elevation of the paretic hemipelvis [8]. Prior work shows both metrics are larger in persons with chronic stroke than in healthy adults during mid-swing [8].

Intervention studies targeting circumduction-related mechanics have yielded inconsistent results. Assistive ankle exosuits have reduced pelvic hike while increasing knee flexion in chronic stroke [9], whereas high-intensity stepping in subacute stroke improved speed and hip range of motion yet increased hip abduction during swing [10]; ankle–foot orthoses have shown no consistent effect on circumduction or pelvic hike [11]. Beyond interventions, mechanical perturbation of pre-swing knee flexion can increase paretic thigh abduction, possibly via abnormal rectus femoris–gluteus medius coupling [12]. Kinematic correlates also appear to vary with walking speed and impairment severity—faster walkers may show greater hip abduction, whereas slower walkers often exhibit reduced knee flexion/dorsiflexion and smaller abduction [13]; pelvic hike is frequently observed with limited paretic knee flexion or more severe paresis [14,15,16]. Collectively, cross-sectional evidence links circumduction surrogates to both segmental motor control and global spatiotemporal context; however, causality and longitudinal behavior remain unresolved [11,14,15,16,17]. Tracking the extent and persistence of these compensatory movements from the acute phase may enable prediction of transitions to chronic maladaptive patterns and the identification of cases requiring early intervention. Consequently, longitudinal studies, particularly in individuals with stroke, are essential for clarifying disease progression and functional changes, evaluating the long-term outcomes of therapeutic interventions, and designing customized physical training programs tailored to individual needs [18,19].

Clinically scalable motion analysis is essential to study these compensations during the acute phase. Markerless depth sensors estimate joint centers from depth images and allow low-cost, preparation-light 3D gait assessment suitable for wards and early rehabilitation [20,21,22,23,24]. Multiple studies—across healthy adults and neurologic populations—support acceptable agreement with optical motion capture for hip and knee kinematics relevant to stance and swing [25,26,27]. Our prior work further demonstrated concurrent validity for thigh abduction and pelvic hike in individuals with hemiparesis after brain injury, supporting the use of a depth sensor to track these compensations in clinical settings [28]. The results of concurrent validity for our three-dimensional motion analysis system and depth sensor were calculated using intraclass correlation coefficients (ICC). Relative agreement (ICC_3,1_) and absolute agreement (ICC_2,1_) were evaluated. For paretic-side pelvic hike: ICC_3,1_ = 0.59, 95%CI (Confidence Interval) (0.17–0.83); ICC_2,1_ = 0.55, 95% CI (0.14–0.81). Circumduction: ICC_3,1_ = 0.92 95%CI (0.79–0.97), ICC_2,1_ = 0.81 95%CI (0.03–0.95), maximum knee flexion angle during swing phase on the paretic side (K5): ICC_3,1_ = 0.86 95%CI (0.65–0.95), ICC_2,1_ = 0.87 95%CI (0.67–0.95).

However, no longitudinal study has quantified how thigh abduction and pelvic hike change over time during the acute phase of stroke recovery, or identified which segmental motor functions are associated with their attenuation [10,13,14,15,16,17]. Addressing this gap could help reframe these behaviors not merely as “abnormalities,” but as adaptive strategies that recede with recovery. The objectives were to quantify short-term longitudinal changes in paretic thigh abduction and pelvic hike during gait in acute stroke, and to identify their associations with lower-limb motor functions. We hypothesized the following: (i) Both thigh abduction and pelvic hike would decrease at follow-up compared with the initial assessment. (ii) Reductions in thigh abduction would be negatively associated with improvements in paretic ankle motor function, and reductions in pelvic hike would be negatively associated with improvements in paretic knee motor function.

## 2. Materials and Methods

### 2.1. Participants

This longitudinal observational study included patients with m admitted to the Departments of Neurology and Neurosurgery at Sendai Medical Center between May 2024 and September 2025. A total of 22 patients were enrolled.

The initial assessment was conducted once the patient was able to walk safely with minimal assistance, and the follow-up assessment was performed 10–14 days later, including gait and physical function evaluations.

During this period, all participants received standard inpatient rehabilitation tailored to their individual clinical needs, as per the standard of care at our institution. This study was observational, and therefore, we did not apply a specific intervention protocol or control the therapy content or intensity.

The inclusion criteria were: (1) patients aged 20–90 years; (2) Diagnosed with first-ever stroke (cerebral infarction or hemorrhage) by a physician based on Magnetic Resonance Imaging (MRI) or Computed Tomography (CT); (3) Ability to walk 3 m with handrail use and minimal assistance; (4) Ability to understand verbal instructions. Exclusion criteria for patients with hemiparesis were: (1) presence of diabetic neuropathy, (2) motor paralysis symptoms in the non-paretic lower limb, (3) decreased sensory function in the non-paretic lower limb, (4) decreased or absent Achilles tendon reflex in the non-paretic side, (5) a history of bone or joint disease in the lower limbs, (6) presence of lesions in the cerebellum or brainstem, (7) symptoms of hemispatial neglect with a score of 2 or lower on the visuospatial cognitive test of the Stroke Impairment Assessment Set (SIAS), and (8) a Mini Mental State Examination (MMSE) score of 19 or lower [29,30,31].

All participants provided written informed consent. The study protocol was approved by the Ethics Committees of Tohoku University Graduate School of Medicine (approval no. 2024-1-032) and Sendai Medical Center (approval no. 24-12) and conducted in accordance with the Declaration of Helsinki.

### 2.2. Measuring Equipment

**Figure 1:**
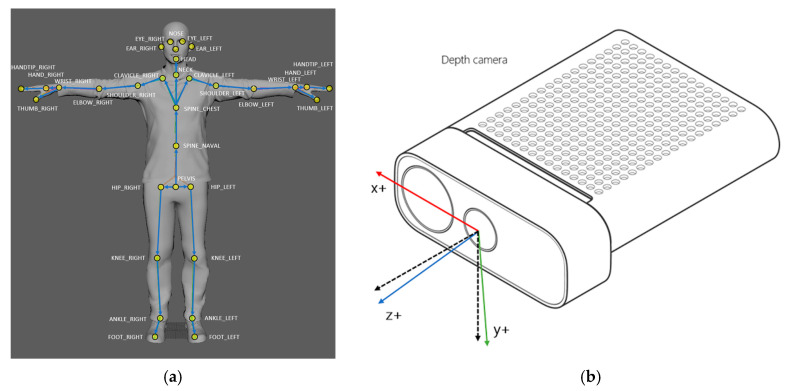
(**a**) Azure Kinect tracks 32 anatomical landmarks [21]; (**b**) The Azure Kinect DK Coordinate System is defined where the x-axis (red) represents the horizontal axis (mediolateral), the y-axis (green) represents the vertical axis, and the z-axis (blue) represents the depth axis (direction of progression) [22]. In addition, the z-axis and y-axis directions of the color camera are specifically indicated by the black dashed lines.

Gait data were collected using a depth sensor (Azure Kinect DK; Microsoft Corporation, Redmond, WA, USA), identical to the setup in our previous validation study [28]. The device captured skeletal joint coordinates at 30 Hz using the Azure Kinect Body Tracking SDK (v1.4.1) (Figure 1a,b) [21,22].

The sensor was mounted on a tripod at a height of 1.0 m, positioned 4.5 m in front of the gait initiation point with a 0° frontal view. The effective measurement range was 0.5–3.86 m [32]. This configuration has previously demonstrated excellent concurrent validity with optical motion capture for hip and knee kinematics (r ≥ 0.9) under comparable conditions [33].

### 2.3. Gait Assessment

Before gait measurement, physical function was assessed using the SIAS.

Participants rolled up their trouser cuffs to expose the ankle and knee joints, tucked in shirts to visualize hip movement, and walked barefoot to improve motion tracking.

**Figure 2:**
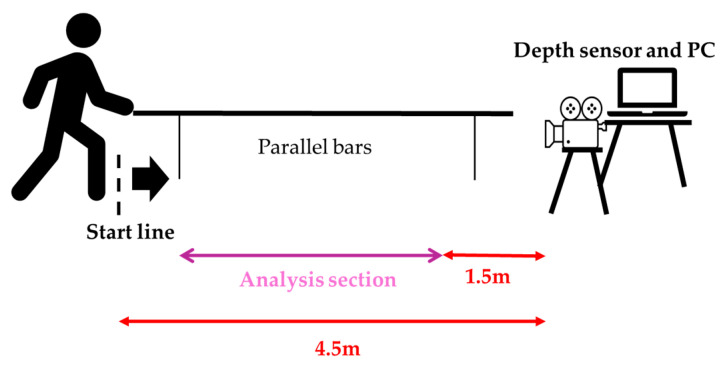
Measurement environment and analysis section.

**Figure 3:**
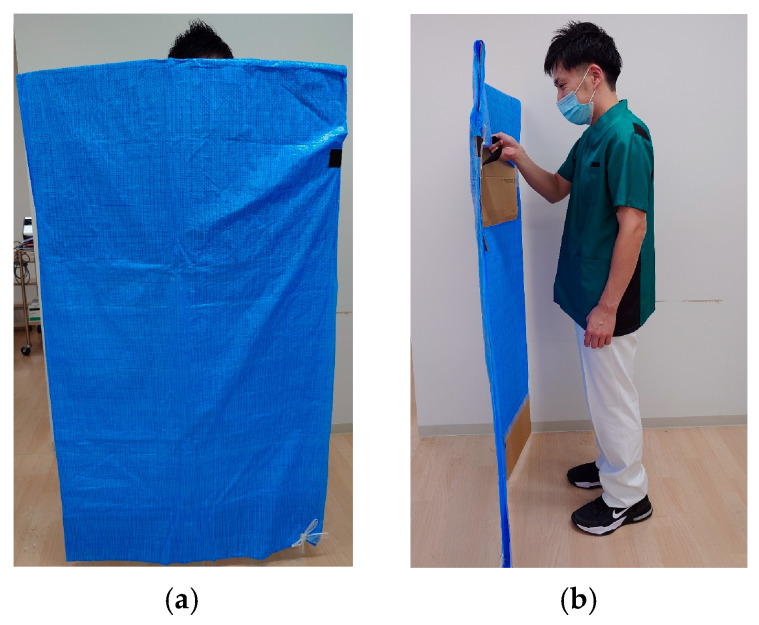
(**a**) The figure shows the evaluator holding a shielding plate as viewed from the frontal plane.; (**b**) This is a diagram showing the evaluator holding a shielding plate as viewed from the sagittal plane.

From a standing start 4 m from the sensor, participants walked 3 m at a self-selected comfortable speed while lightly holding a handrail with the non-paretic upper limb (Figure 2). Handrail use was mandatory during this task. When minimal assistance was required, the evaluator used a cardboard shield to prevent self-detection by the sensor and provided minimal manual support under the paretic axilla (Figure 3).

Each participant completed five valid trials (maximum of eight, including repetitions). Rest periods were allowed to avoid fatigue. If a trial was invalid (e.g., tracking loss), it was immediately repeated. To ensure consistency across sessions, handrail and orthosis use were standardized: patients who used a handrail or ankle–foot orthosis (AFO) during the initial test used the same aid during the follow-up assessment.

### 2.4. Data Processing

Gait analysis processing was performed using Python (version 3.10, Python Software Foundation, Wilmington, DE, USA). The time-series coordinate data obtained from the depth sensor were resampled to 60 Hz and smoothed using a fourth-order Butterworth low-pass filter with a 6 Hz cutoff frequency. This resampling strategy is supported by a focused review of Kinect gait validation, which reported that upsampling Kinect data yields good agreement in key spatiotemporal variables, particularly in studies operating at sampling frequencies of 60–120 Hz [34].

Gait events (initial contact and toe-off) were detected using the depth sensor data. Initial contact was defined as the point of maximum anteroposterior distance between the pelvis and ankle points. Toe-off was defined as the point of maximum anteroposterior distance between the pelvis and foot points [35].

**Figure 4:**
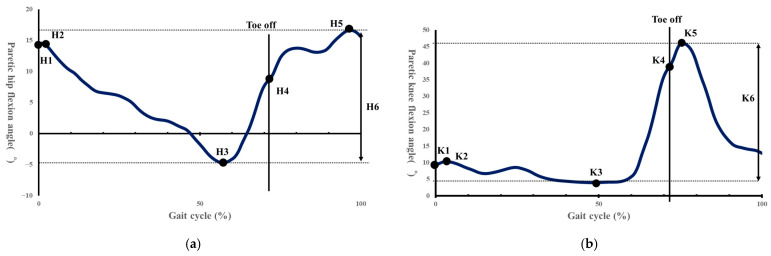
(**a**) Indicates the hip flexion angle during one gait cycle. H1, hip angle at initial contact; H2, maximum flexion angle during loading response; H3, maximum extension angle in stance phase; H4, hip angle at toe off; H5, maximum flexion angle in swing; H6, total sagittal plane excursion [37]. (**b**) Shows the knee joint flexion angle during one gait cycle. K1, knee angle at initial contact; K2, maximum flexion angle during loading response; K3, maximum extension in stance phase; K4, knee angle at toe off; K5, maximum flexion angle in swing; K6, total sagittal plane excursion [37].

**Figure 5:**
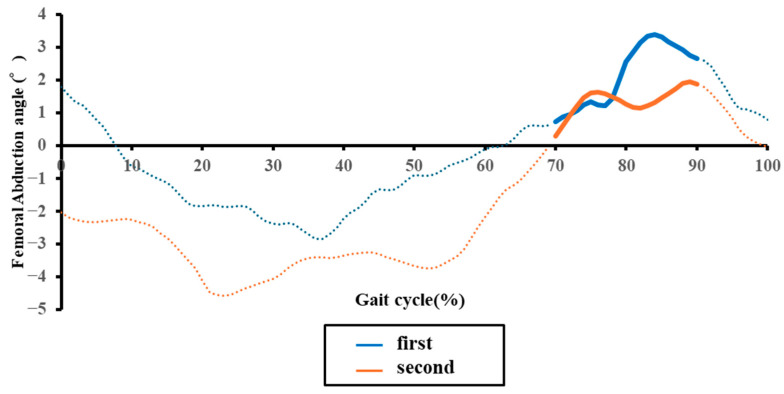
Representative temporal changes in the paretic femoral abduction angle during one gait cycle in an acute stroke patient. The blue and orange lines indicate data from the initial and second assessments, respectively. Solid line segments represent the mid-swing phase, while dashed line segments represent other phases.

Spatiotemporal parameters were calculated according to the methods of Rose et al. [36]. Based on previous studies, kinematic parameters were calculated, including: H1 (hip flexion angle at initial contact), H2 (hip flexion angle during loading response), H3 (minimum hip flexion angle), H4 (hip flexion angle at toe-off), H5 (maximum hip flexion angle during swing), H6 (total hip sagittal plane excursion), K1 (knee flexion angle at initial contact), K2 (knee flexion angle during loading response), K3 (minimum knee flexion angle), K4 (knee flexion angle at toe-off), K5 (maximum knee flexion angle during swing), K6 (total knee sagittal plane excursion), maximum paretic femoral abduction angle, and maximum paretic pelvic hike angle during 25–75% of the paretic swing phase (Figure 4a,b and Figure 5) [8,37].

The paretic femoral abduction angle was defined as the angle formed by the vertical axis of the laboratory and the line connecting the hip and knee joints. Paretic pelvic hike was defined as the angle formed by the horizontal axis of the laboratory and the line connecting left and right hip joints. Furthermore, a positive value was defined when the paretic hip joint was higher than the non-paretic hip joint, and a negative value when it was lower. Our previous research on the concurrent validity between 3D motion analysis and the depth sensor in hemiplegic gait confirmed moderate or higher concurrent validity for these kinematic parameters, excluding H4, H5, and K1 [29].

### 2.5. Statical Analysis

First, the Shapiro–Wilk test was used to assess the normality of distribution for all continuous variables, including gait parameters. To examine the relationship between baseline paretic femoral abduction and pelvic hike angles and other gait and functional parameters, either Pearson’s product-moment correlation coefficient or Spearman’s rank correlation coefficient was used, depending on the data distribution. Comparisons of gait parameters between the initial and second assessments were conducted. A paired *t*-test was used for normally distributed continuous variables, while the Wilcoxon signed-rank test was used for variables that were not normally distributed. Correlations between the changes in paretic femoral abduction and pelvic hike angles and the changes in other gait and functional parameters were also analyzed using Pearson’s or Spearman’s correlation.

The a priori power analysis, using G*Power software (Ver. 3.1) and based on the femoral abduction angle as the main outcome, indicated that 24 participants would be required to detect an expected effect size of d = 0.6 with 80% power (1 − β = 0.80) at a two-tailed significance level of α = 0.05. We therefore aimed to recruit a final sample size of *n* = 24 participants. Given that our actual sample size of 22 participants was slightly below the calculated target, we calculated and reported the effect size (Cohen’s dz) to better quantify the magnitude of the results, in addition to significance testing. Correlation coefficients were interpreted as follows: 0.80 or higher as “very strong”; 0.50 to less than 0.80 as “moderate”; 0.30 to less than 0.50 as “somewhat weak”; and less than 0.30 as “weak” [38]. All statistical analyses were performed using SPSS Statistics version 20.0 (IBM, Armonk, NY, USA). The significance level was set at 0.05.

## 3. Results

### 3.1. Participant Characteristics

Twenty-two patients with first-ever acute stroke were enrolled (17 male; mean age 66.05 years, SD 15.44). The mean time from stroke onset to the initial assessment was 8.77 days (SD 5.27). The mean longitudinal study period (interval between assessments) was 12.95 days (SD 1.15). Mean daily physical therapy time was 22.06 min (SD 7.05) before the initial assessment and 25.72 min (SD 7.68) during the interval (Table 1).

### 3.2. Longitudinal Changes in Gait Parameters

From baseline to follow-up, spatiotemporal parameters showed significant improvement. Gait speed, paretic stride length, and step lengths increased, while gait cycle time, double support time, and paretic stance time decreased (all *p* < 0.01; Table 2).

**Figure 6:**
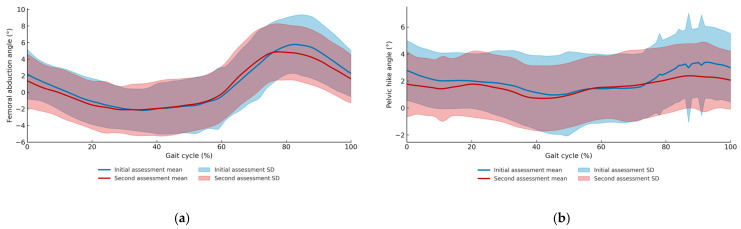
(**a**) Mean and standard deviation of femoral abduction angles across the gait cycle for the first and second assessments. (**b**) Mean and standard deviation of pelvic hike angles across the gait cycle for the first and second assessments.

Regarding the kinematic variables of clinical interest, compensatory angles declined significantly: thigh abduction decreased (mean ± SD: 6.71 ± 3.78° to 5.79 ± 3.47°; *p* = 0.049), as did pelvic hike (3.82 ± 2.34° to 2.90 ± 2.35°; *p* = 0.025) (Figure 6a,b). Concurrently, segmental kinematics improved, with significant increases in total hip sagittal plane excursion (H6) (*p* = 0.039), maximum knee flexion in swing (K5) (*p* = 0.026), and total knee sagittal plane excursion (K6) (*p* = 0.036) (Table 2).

### 3.3. Longitudinal Changes in Measures of Physical Function

SIAS motor scores improved at follow-up for the hip (*p* < 0.001), knee (*p* = 0.006), and ankle (*p* < 0.001), as did deep sensation (*p* = 0.046) and abdominal muscle strength (*p* = 0.002) (Table 3).

### 3.4. Associations Between Changes in Compensatory Movements and Motor Function

On the paretic side, the mean change (±SD) was −0.92 ± 2.06° for femoral abduction and −0.91 ± 1.74° for pelvic hike. The reductions in these two compensatory movements were positively correlated (r = 0.55, *p* = 0.008) (Table 4).

Regarding motor recovery, the reduction in thigh abduction showed a significant negative correlation with improvement in paretic ankle motor function (ρ = −0.49, *p* = 0.021). Similarly, the reduction in pelvic hike was negatively correlated with improvement in paretic knee motor function (ρ = −0.43, *p* = 0.046) (Table 5).

## 4. Discussion

This longitudinal study quantified short-term changes in paretic femoral abduction and pelvic hike during gait in acute post-stroke hemiparesis and examined their associations with segmental motor recovery. At baseline, greater thigh abduction was linked to poorer paretic ankle motor function and wider step width, whereas greater pelvic hike was linked to slower and more asymmetrical spatiotemporal patterns and to poorer knee/hip motor function. Over approximately 13 days, both compensatory angles decreased, and their reductions were negatively associated with improvements in ankle (for thigh abduction) and knee (for pelvic hike) motor function, respectively. Taken together, these findings support the view that these behaviors are distinct compensatory strategies that tend to recede as segmental control recovers, especially at the ankle and knee.

### 4.1. Relationship Between Compensatory Movements and Gait/Physical Function at Initial Assessment

A At the initial assessment, greater thigh abduction co-occurred with poorer ankle motor function (SIAS) and wider step width. This pattern is consistent with a lateral stability strategy when distal control is compromised: stroke survivors have reduced accuracy of hip abduction and tend to widen step width to stabilize the stance phase [39]. In contrast, pelvic hike aligned with slower gait and longer double-support, stance, and swing times—patterns characteristic of more severe lower-limb paresis [40]. This pattern is consistent with the interpretation that pelvic hike may be associated with compensatory strategies used to manage functional limb shortening resulting from limited knee flexion [41].

Little et al. classified post-stroke patients by vertical and lateral pelvic displacement and reported that those with greater displacement had poorer lower-limb motor function and greater asymmetry in hip extension and ankle plantarflexion [42]. These individuals generated less paretic propulsion in late stance, resulting in reduced knee flexion in swing and greater reliance on pelvic hike to ensure foot clearance. The present findings are consistent with this compensatory pattern observed in more severe cases, reflecting an adaptive strategy to maintain foot clearance and stance stability when paretic propulsion and knee flexion are limited.

### 4.2. Longitudinal Changes in Gait: Context for Compensation Attenuation

Across the cohort, gait speed, stride length, and step length increased, whereas gait cycle time, double-support time, and paretic stance time decreased. These changes coincide with improvements in hip, knee, and ankle motor function and range of motion, suggesting a transition toward more typical gait patterns as paresis recovers.

Similar trends have been reported during 2–12 months post-stroke: gait speed and step length increase alongside rising Fugl-Meyer lower-limb scores, reflecting parallel gains in walking ability and motor recovery [43,44]. Continuous gait assessments during hospitalization have also linked increased trunk acceleration regularity and improved lower-limb extension in late stance with enhanced gait speed [45,46].

However, greater speed does not always signify true paretic-side recovery—improvement may arise from non-paretic propulsion [47]. Hence, frameworks distinguishing quantitative recovery (speed gain) from qualitative recovery (paretic-side functional return) have been proposed, emphasizing paretic propulsion as a key indicator [48]. In this context, the observed increases in speed and reductions in double-support time occurred alongside decreases in compensatory behavior, suggesting that these motor improvements and compensatory patterns may change in parallel over time.

### 4.3. Temporal Changes in Paretic Femoral Abduction Angle and Its Relationship with Physical Function

**Figure 7:**
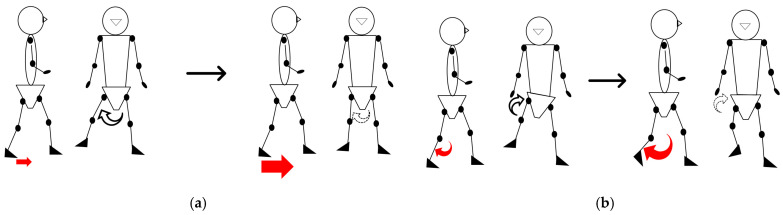
The figure illustrates the relationships between motor function deficits and compensatory strategies: (**a**) The relationship between reduced ankle motor function, resulting in poor propulsion (indicated by the small arrows), and the increased reliance on femoral abduction; and (**b**) the relationship between reduced knee motor function, causing coordination difficulty (indicated by the small arrows), and the increased reliance on pelvic hike.

The paretic femoral abduction angle decreased over time and showed a significant negative association with improvements in paretic ankle motor function. This observed relationship is consistent with two interacting mechanisms that accompany distal motor recovery: Firstly, Propulsive Recovery: Improved plantar flexor activity enhances late-stance push-off, potentially reducing the demand for lateral abduction required to achieve swing-phase clearance. Secondly, Coordination Changes: Abnormal reflex coupling between the rectus femoris and gluteus medius can promote excessive abduction when ankle propulsion is weak [12,49]. As ankle control improves, this abnormal synergy may diminish, consequently lessening the need for lateral compensation (Figure 7a).

Thus, the attenuation of thigh abduction reflects both mechanical sufficiency and coordination refinement—a biomechanical pattern that accompanies distal motor recovery and warrants further investigation into its causal role.

### 4.4. Temporal Changes in Paretic Pelvic Hike and Its Relationship with Physical Function

Pelvic hike also decreased, and its change magnitude correlated negatively with improvement in paretic knee motor control. Interestingly, the pelvic hike was more strongly related to knee control than to static flexion amplitude, implying that hike compensates not just for limited knee flexion angle but for difficulties coordinating flexion–extension timing (Figure 7b).

Previous research showed that functional limb shortening depends on both hip and knee contributions [14], and that circumduction cannot be fully explained by reduced knee flexion or ankle dorsiflexion alone [14,16]. Akbas et al. also reported that hip circumduction is not simply a substitute for limited knee flexion, emphasizing the role of intersegmental coordination [14]. Our results—linking pelvic hike more to motor function than to angle magnitude—concur with this view.

Additionally, our previous validation study, which assessed the concurrent validity between a 3D motion analysis and a depth sensor in this population, revealed proportional bias in the maximum paretic knee flexion angle during the swing phase [28]. This measurement limitation may have weakened the angle-based correlations but does not negate the stronger link to motor recovery. Altogether, improvements in knee control and smoother foot clearance were observed together with reduced pelvic elevation, indicating parallel changes in these parameters rather than a directional causal relationship.

### 4.5. Limitations and Future Research

This study has several limitations. First, because gait was assessed within parallel bars with mandatory handrail support, upper-limb loading and altered balance demands may have influenced lower-limb kinematics. Although this setup ensured participant safety, it may reduce ecological validity compared with overground walking without external support. Therefore, the generalizability of the findings to unsupported gait should be interpreted with caution.

Second, this study did not include measurements for the trunk and non-paretic lower limbs. These components were not evaluated due to the low concurrent validity of the depth sensor, a potential confounding factor. This omission is significant, as compensatory movements of the non-paretic lower limb were not fully considered. Stroke patients may compensate for issues such as foot clearance by using non-paretic ankle plantarflexion or hip extension, and the extent of these strategies could alter the magnitude of paretic-side compensation [16]. Therefore, combining a quantitative evaluation of non-paretic kinematic and kinetic data would be necessary to clarify the overall picture of compensatory movements.

Third, the physical therapy content and frequency during the observation period were not standardized. Compensatory movement trends may vary depending on the specific therapeutic interventions. Although the average daily provision of physical therapy was approximately 25 min, variability in therapy content and frequency may still influence gait changes. Thus, intervention effects could not be fully controlled in this study.

Fourth, due to the characteristics of the depth sensor used, a proportional error exists in the paretic knee flexion angles. Our previous validation study confirmed that the maximum paretic knee flexion angle during the swing phase exhibits a significant proportional bias. Specifically, the regression analysis confirmed a significant proportional error (Slope = 0.348, 95% CI [0.11, 0.59], *p* = 0.007), and the Limits of Agreement (LoA) were in the range of −15.176 to 14.026 [28]. Using an optical 3D motion analysis system would improve joint angle accuracy and enable a more precise evaluation of changes over time.

Fifth, we did not perform a kinetic assessment and thus did not directly examine kinetic factors such as propulsion or ground reaction forces. While kinetic evaluation poses challenges regarding safety and equipment constraints in acute patients, future research should consider introducing simplified measurement methods using wearable sensors or force plates.

Sixth, ankle joint angles were not quantitatively assessed. It is possible that recovery of paretic ankle dorsiflexion and plantarflexion improved foot clearance, consequently contributing to the decrease in paretic femoral abduction. Future studies should conduct multi-joint analyses including ankle angles to elucidate the factors behind compensatory movements in greater detail.

Seventh, lesion location and volume, known contributors to gait recovery, were not accounted for in this dataset [50].

Eighth, we also did not control for walking aids or orthotic use, which may alter limb kinematics and compensatory strategies.

The present findings provide several practical implications for therapists and practitioners working with individuals after stroke. Identifying circumduction during gait can help clinicians determine whether this compensatory movement reflects underlying deficits such as reduced knee flexion and impaired motor control. Based on this assessment, therapists can select targeted interventions—such as task-specific gait training, knee flexion facilitation and assistive device adjustments—to reduce excessive circumduction when it is maladaptive. Conversely, when circumduction functions as an effective temporary strategy, clinicians may choose to tolerate it while addressing more fundamental impairments. Integrating this clinical reasoning into patient education may enhance shared decision-making and promote individualized rehabilitation planning [51].

## 5. Conclusions

This study longitudinally investigated paretic femoral abduction and pelvic hike angles during gait in patients with acute stroke hemiparesis and clarified the relationship between these angles and lower limb motor function.

The results showed that both the paretic femoral abduction angle and paretic pelvic hike angle significantly decreased over time, indicating a reduction in compensatory movements during gait. Furthermore, the improvement in paretic ankle motor function showed a significant negative correlation with the decrease in the femoral abduction angle, and the improvement in paretic knee motor function showed a significant negative correlation with the decrease in the pelvic hike angle.

These results suggest that in these patients, the recovery of paretic ankle and knee motor functions leads to a reduction in compensatory paretic femoral abduction and pelvic hike during gait. In other words, this indicates that as the functional propulsive force and knee flexion/extension control ability of the paretic lower limb recover, the dependence on compensatory movements such as femoral abduction and pelvic hike naturally decreases.

## Figures and Tables

**Table 1:** Characteristics of stroke patients.

	Stroke Patients	
Gender (male/female) ^a^	17/5	
Age (year) ^b^	66.05	(15.44)
Height (m) ^b^	1.65	(0.08)
Weight (kg) ^b^	68.43	(15.43)
Diagnosis (Hemorrhage/Infarction) ^a^	5/17	
Paretic side (left/right) ^a^	14/8	
Time since onset (days) ^b^	8.77	(5.27)
Longitudinal study period	12.95	(1.15)
Physical therapy time during longitudinal period (minutes)	25.72	(7.68)

^a^ Number of people. ^b^ Mean (Standard deviation).

**Table 2:** Changes in gait parameters over time.

Spatiotemporal Parameters	Initial Assessment	Second Assessment	Effect Size	*p*-Value
Gait speed (m/s) ^a^	0.38 (0.16)	0.53 (0.18)	−1.18	<0.001
Double stance time (s) ^b^	0.49 (0.23)	0.35 (0.18)	0.88	<0.001
Paretic stance time (s) ^a^	1.44 (0.54)	1.10 (0.34)	−0.79	<0.001
Paretic swing time (s) ^b^	0.53 (0.16)	0.48 (0.11)	−0.42	0.051
Stride length on the paretic side (m) ^a^	0.66 (0.15)	0.78 (0.14)	−1.08	<0.001
Step length on the paretic side (m) ^a^	0.31 (0.08)	0.36 (0.07)	−0.72	0.003
Step width (m) ^a^	0.13 (0.03)	0.12 (0.04)	0.30	0.174
Paretic femoral abduction angle (°) ^a^	6.71 (3.78)	5.79 (3.47)	0.51	0.049
Paretic pelvic hike angle (°) ^a^	3.82 (2.34)	2.90 (2.35)	0.51	0.025
H1 (°) ^b^	23.18 (5.67)	23.93 (4.30)	−0.17	0.424
H2 (°) ^a^	23.20 (5.40)	24.02 (4.29)	−0.20	0.367
H3 (°) ^a^	−3.89 (6.17)	−5.91 (5.13)	0.42	0.064
H4 (°) ^a^	5.52 (7.12)	4.69 (5.76)	0.14	0.521
H5 (°) ^a^	24.28 (6.79)	25.55 (5.49)	−0.25	0.263
H6 (°) ^a^	29.59 (5.42)	32.32 (5.43)	−0.47	0.039
K1 (°) ^a^	9.93 (5.16)	8.58 (5.23)	0.34	0.124
K2 (°) ^a^	15.15 (4.80)	15.09 (6.20)	0.01	0.963
K3 (°) ^a^	5.65 (3.16)	5.96 (4.40)	−0.13	0.543
K4 (°) ^a^	35.24 (10.95)	37.74 (8.89)	−0.36	0.108
K5 (°) ^a^	46.65 (11.67)	50.87 (10.82)	−0.51	0.026
K6 (°) ^a^	41.82 (10.73)	45.84 (9.77)	−0.48	0.036

Mean (Standard deviation). ^a^ Paired *t*-test (*p* < 0.05). ^b^ Wilcoxon signed-rank test (*p* < 0.05).

**Table 3:** Changes in physical function assessment over time.

SIAS Evaluation Items	First Assessment	Second Assessment	Effect Size	*p*-Value
Hip joint (0/1/2/3/4/5) ^a^	3 (4-2)	4 (5-3)	−0.82	<0.001
Knee joint (0/1/2/3/4/5) ^a^	4 (4-3)	4 (5-3)	−0.58	0.006
Ankle joint (0/1/2/3/4/5) ^a^	3 (4-2)	4 (5-3)	−0.74	<0.001
L/E Deep tendon reflex (0/1/2/3) ^a^	1 (2-1)	2 (2-1)	−0.30	0.153
L/E muscle tone (0/1/2/3) ^a^	2 (3-2)	3 (3-2)	−0.30	0.160
Superficial sensation (0/1/2/3) ^a^	3 (3-3)	3 (3-3)	−0.37	0.083
Deep sensation (0/1/2/3) ^a^	3 (3-3)	3 (3-3)	−0.42	0.046
Abdominal strength (0/1/2/3) ^a^	1 (2-0)	2 (3-2)	−0.66	0.002

SIAS: Stroke impairment assessment set (Interquartile range). L/E: Lower extremity. ^a^ Wilcoxon signed-rank test (*p* < 0.05).

**Table 4:** Correlations between changes in compensatory movements and gait parameters.

Spatiotemporal and Kinematic Variables	Mean (SD)	Paretic Side Femoral Abduction Angle			Paretic Side Pelvic Hike Angle		
		Correlation	*p*-Value	1 − β	Correlation	*p*-Value	1 − β
Gait speed Δ (m/s) ^a^	0.15 (0.13)	0.09	0.701	0.94	0.03	0.886	0.97
Double stance time Δ (s) ^a^	−0.14 (0.16)	−0.24	0.290	0.81	0.12	0.609	0.92
Paretic stance time Δ (s) ^b^	−0.34 (0.34)	−0.19	0.393	0.87	0.10	0.661	0.94
Paretic swing time Δ (s) ^b^	−0.05 (0.13)	0.32	0.148	0.70	0.41	0.058	0.52
Paretic stride length Δ (m) ^a^	0.12 (0.11)	0.03	0.881	0.97	0.00	0.996	0.98
Paretic step length Δ (m) ^a^	0.04 (0.06)	−0.03	0.892	0.97	0.15	0.517	0.90
Step width Δ (m) ^a^	−0.01 (0.03)	−0.22	0.332	0.84	0.12	0.604	0.92
Paretic pelvic hike Δ (°) ^a^	−0.91 (1.74)	0.55	0.008	0.23	-	-	
H1Δ (°) ^b^	0.75 (4.33)	−0.31	0.154	0.71	−0.32	0.148	0.70
H2Δ (°) ^a^	0.83 (4.20)	−0.34	0.118	0.66	−0.27	0.221	0.77
H3Δ (°) ^a^	−2.02 (4.84)	−0.03	0.886	0.97	−0.14	0.545	0.91
H4Δ (°) ^a^	−0.83 (5.99)	0.02	0.938	0.97	−0.14	0.540	0.91
H5Δ (°) ^a^	1.27 (5.19)	−0.13	0.557	0.92	−0.13	0.557	0.92
H6Δ (°) ^a^	2.73 (5.82)	−0.09	0.679	0.94	0.02	0.936	0.97
K1Δ (°) ^a^	−1.35 (3.94)	−0.09	0.690	0.94	−0.25	0.271	0.80
K2Δ (°) ^a^	−0.06 (5.98)	−0.23	0.311	0.83	−0.13	0.569	0.92
K3Δ (°) ^a^	0.31 (2.38)	−0.36	0.096	0.62	−0.22	0.324	0.84
K4Δ (°) ^a^	2.49 (6.96)	0.26	0.247	0.79	0.03	0.894	0.97
K5Δ (°) ^a^	4.22 (8.24)	0.1	0.660	0.94	0.09	0.700	0.94
K6Δ (°) ^a^	4.02 (8.40)	0.19	0.388	0.87	0.13	0.573	0.92

SD: Standard deviation. ^a^ Pearson’s correlation coefficient (*p* < 0.05). ^b^ Spearman’s rank correlation coefficient (*p* < 0.05).

**Table 5:** Correlations between changes in compensatory movements and physical function assessment items.

SIAS Evaluation Items	Median (IQR)	Paretic Side Femoral Abduction Angle			Paretic Side Pelvic Hike Angle		
		Correlation	*p*-Value	1 − β	Correlation	*p*-Value	1 − β
Hip joint Δ ^b^	1 (1-1)	0.42	0.050	0.50	−0.20	0.371	0.86
Knee joint Δ ^b^	0 (1-0)	−0.28	0.203	0.76	−0.43	0.046	0.48
Ankle joint Δ ^b^	1 (1-0)	−0.49	0.021	0.35	−0.01	0.962	0.97
L/E deep tendon reflex Δ ^b^	0 (1-0)	−0.34	0.121	0.66	−0.13	0.566	0.92
L/E muscle tone Δ ^b^	0 (0)	0.01	0.969	0.66	−0.31	0.161	0.71
L/E superficial sensation Δ ^b^	0 (0)	−0.16	0.487	0.97	−0.03	0.888	0.97
L/E deep sensation Δ ^b^	0 (0)	0.02	0.935	0.90	0.13	0.559	0.92

SIAS: Stroke Impairment Assessment Set. IQR: Interquartile range. L/E: Lower extremity. ^b^ Spearman’s rank correlation coefficient (*p* < 0.05).

## Data Availability

The data, including graphs, within this paper are available from the corresponding author upon reasonable request.
